# Molting in Pancrustacea Is Characterized by Both Deeply Conserved and Recently Evolved Gene Modules

**DOI:** 10.1093/gbe/evag212

**Published:** 2026-08-24

**Authors:** Kenneth Kim, Giulia Campli, Sagane Joye, Ariel D Chipman, Robert M Waterhouse, Marc Robinson-Rechavi

**Affiliations:** Department of Ecology and Evolution, University of Lausanne, Lausanne 1015, Switzerland; SIB Swiss Institute of Bioinformatics, Lausanne 1015, Switzerland; Department of Ecology and Evolution, University of Lausanne, Lausanne 1015, Switzerland; SIB Swiss Institute of Bioinformatics, Lausanne 1015, Switzerland; Department of Ecology and Evolution, University of Lausanne, Lausanne 1015, Switzerland; SIB Swiss Institute of Bioinformatics, Lausanne 1015, Switzerland; Department of Ecology, Evolution and Behavior, The Silberman Institute of Life Science, The Hebrew University of Jerusalem, Jerusalem, Israel; SIB Swiss Institute of Bioinformatics, Lausanne 1015, Switzerland; Department of Ecology and Evolution, University of Lausanne, Lausanne 1015, Switzerland; SIB Swiss Institute of Bioinformatics, Lausanne 1015, Switzerland

**Keywords:** arthropods, molting, evo-devo, gene expression, evolutionary genomics

## Abstract

Arthropods such as insects and crustaceans, which together form the monophyletic group Pancrustacea, possess a rigid chitinous exoskeleton that must be periodically shed through molting to allow growth and morphological change. Although molting is a deeply conserved developmental process across Arthropoda, our understanding of its molecular mechanisms is still largely derived from insect model species. Lineage-specific innovations and losses of molting-related genes raise fundamental questions about the extent of its conservation outside noninsect arthropods. Here, we investigate the evolutionary conservation of molting gene expression across five representative pancrustacean species using publicly available transcriptomic datasets. Changes in gene expression during molting are characterized by both deeply conserved and lineage-specific gene modules. Temporal gene expression analyses reveal that these lineage-specific signatures are not uniformly distributed across the molting process: the middle transitional phase is more lineage-specific, thereby exhibiting an inverse hourglass pattern. This is likely due to life-history-specific processes, development of the cuticle, and specialized structures of the exoskeleton. Overall, this study provides evidence for both the evolutionary conservation and divergence of this key postembryonic developmental process and highlights the modular architecture of the molting program.

Significance Insects and crustaceans are very diverse, yet share a fundamental need to molt. This molting enable growth, and sometimes dramatic changes during metamorphosis of some insects. We show that some genes active in molting are shared between these species, while most are different. Yet the different genes control shared processes, which enable the change of exoskeleton and the necessary physiological changes.

## Introduction

The phylum Arthropoda includes a vast array of extant and extinct taxa, such as insects, crustaceans, arachnids, trilobites, and sea scorpions. These organisms share defining features: segmented bodies, paired jointed appendages, and a chitin-based exoskeleton, often reinforced with proteins or minerals (R.F. Chapman et al. 2013; Daley et al. 2018; Giribet and Edgecombe 2019). The modular organization of their segmented body plan, combined with a protective exoskeleton, has enabled arthropods to thrive in an extraordinary range of ecological niches. However, their rigid exoskeleton also hinders their ability to grow and must be periodically shed in a process called molting, which not only enables growth but also facilitates morphological changes during development (R.F. Chapman et al. 2013; Truman 2019; Truman and Riddiford 2019; Belles 2020).

The molting process is governed by hormonal regulation, primarily through ecdysteroids and sesquiterpenoids (Cheong et al. 2015; Qu et al. 2018; Truman and Riddiford 2019; Campli et al. 2024). Hormones are synthesized in secretory organs in response to signals from neuropeptides. Once released into the hemolymph, these hormones reach target tissues, where they trigger transcription factors responsible for various molting pathways (Truman 2019). As hormone levels decline, neurosecretions activate the “ecdysis motor”, a behavioral sequence that leads to the final stages of molting and exoskeleton hardening (White and Ewer 2014; Roer et al. 2015; Zieger et al. 2021).

Molting is coordinated by the increase and decrease of ecdysteroid hormone or its active form 20-hydroxyecdysone (20E) (Truman and Riddiford 2019) and occurs in distinct molt stages each characterized by different morphological and behavioral changes (Drach and Tchernigovtzeff 1967; Mykles 2011; Gorissen and Sandeman 2022; Kim et al. 2024). During the pre-molt phase, enzymes digest the inner layers of the old cuticle, allowing the exoskeleton to detach from the epidermal cells a process known as apolysis (Roer et al. 2015; Zhang et al. 2021). Molting sensu stricto, or ecdysis, involves the rupturing of the old exoskeleton due to body movements and increased hemolymph pressure, enabling the animal to shed the exuvia (Daley and Drage 2016). In the post-molt phase, the new exoskeleton hardens through processes like sclerotization, mineralization, and pigmentation, which collectively transform the soft cuticle into a mature and hardened exoskeleton.

Most of our knowledge of the molecular and biochemical mechanisms underlying molting has been derived from a small number of insect model species, such as *Tribolium castaneum*, *Bombyx mori*, or *Drosophila melanogaster* (Truman and Riddiford 2019; Campli et al. 2024). As a consequence, molecular insights into molting largely stem from developmental contexts associated with insect metamorphosis, despite molting itself being a shared and ancient process across Arthropoda and our understanding of molting beyond Insecta remains limited (Campli et al. 2024). The increasing availability of genomic resources and sequencing data from a broader range of arthropods, driven in part by large-scale projects such as the i5k initiative (i5K Consortium 2013) and the European Reference Genome Atlas (ERGA) (Mazzoni et al. 2023), has enabled broader testing of the involvement of genes in molting that were first identified in insect model species (Campli et al. 2024). These genomic analyses have confirmed the presence of a conserved core set of genes involved in molting, found across various lineages (Campli et al. 2024; Campli et al. 2026). While a conserved genetic backbone for molting exists, lineage-specific modifications are evident such as the incomplete ecdysteroid biosynthetic pathway in barnacles (Dermauw et al. 2020) and the missing *Shd* (*cyp314a1*) gene in several fungus-farming ant species (Dermauw et al. 2020). Additional examples of losses include *neverland* in Varroa mites (Cabrera et al. 2015; Techer et al. 2019; Campli et al. 2026) and in beetles (Campli et al. 2025; Perry and Robin 2025), *phantom* (*phm*) in chelicerates (Schumann et al. 2018), and *juvenile hormone acid methyltransferase* (*JHAMT*) gene in millipedes (So et al. 2022). Various genes also appear to be restricted to certain clades such as *Nobo* which is only found in Diptera and Lepidoptera (Enya et al. 2015; Koiwai et al. 2020), or *spok* and *Cyp6t3* found only in Drosophilidae (Ono et al. 2006; Sztal et al. 2007; Ou et al. 2011). Collectively, these patterns show that even key genes regulating molting are not all equally conserved. The conservation of the broader set of genes involved in molting is not well known. This is partly due to the candidate gene approach commonly used in functional studies, which prioritizes known genes from model organisms and thus leads to a systematic bias toward studying conserved genes. As most genes function within biological pathways or coregulated modules (Oleksiak et al. 2002; Brawand et al. 2011; Harrison et al. 2012; Keidar Haran and Keren 2022), investigating the expression patterns of genes during molting is essential to understand the conservation and evolution of molting processes across Arthropoda.

Phylogenetic analysis suggests that the hexapod clade, which includes insects, represents an ancient divergence within Pancrustacea, indicating that insects evolved from crustacean-like ancestors (Rota-Stabelli et al. 2013; Wolfe et al. 2016; Schwentner et al. 2017; Bernot et al. 2023). This diversity makes Pancrustacea an interesting group for studying the conservation and divergence of molting mechanisms. Molting in insects and crustaceans is orchestrated by neuropeptide-driven endocrine cascades regulated by specialized steroidogenic glands, the prothoracic gland (PG) in insects and the Y-organ (YO) in crustaceans (Cheong et al. 2015; Truman and Riddiford 2019; Mykles and Chang 2020; Mykles 2021). In insects, molting is triggered by the release of prothoracicotropic hormone, which binds to the Torso receptor on the PG and activates the mitogen-activated protein kinase (MAPK) and mammalian target of rapamycin (mTOR) pathways, leading to the production of ecdysteroids (Truman and Riddiford 2019). Both larval-to-larval and metamorphic molts are regulated by conserved endocrine pathways involving ecdysteroids and juvenile hormone (JH), with larval identity maintained by *chinmo* and metamorphic transitions associated with the sequential activation of *broad* and *E93* (Truman and Riddiford 2026). In contrast, crustaceans rely on inhibitory control: molt-inhibiting hormone (MIH), secreted from the eyestalk X-organ/sinus gland complex, suppresses YO activity through a cAMP/Ca^2+^- and NO/cGMP-dependent signaling cascade (Mykles 2011). Reduction of MIH release, either through environmental cues or experimental eyestalk ablation, activates the YO and triggers molting. In addition, the calcium-rich marine environment has enabled crustaceans to build calcified, rigid exoskeletons that provide strong protection. In terrestrial environments, where calcium is less readily available, insects have adopted a different strategy by developing lighter, more flexible cuticles that rely on biochemical processes such as laccase-mediated cross-linking for cuticle hardening and tanning (Asano et al. 2019; Volovych et al. 2025). These differences in molting mechanisms and exoskeletal properties offer a comparative framework for exploring the evolutionary conservation of molting across Pancrustacea.

**Fig. 1.**
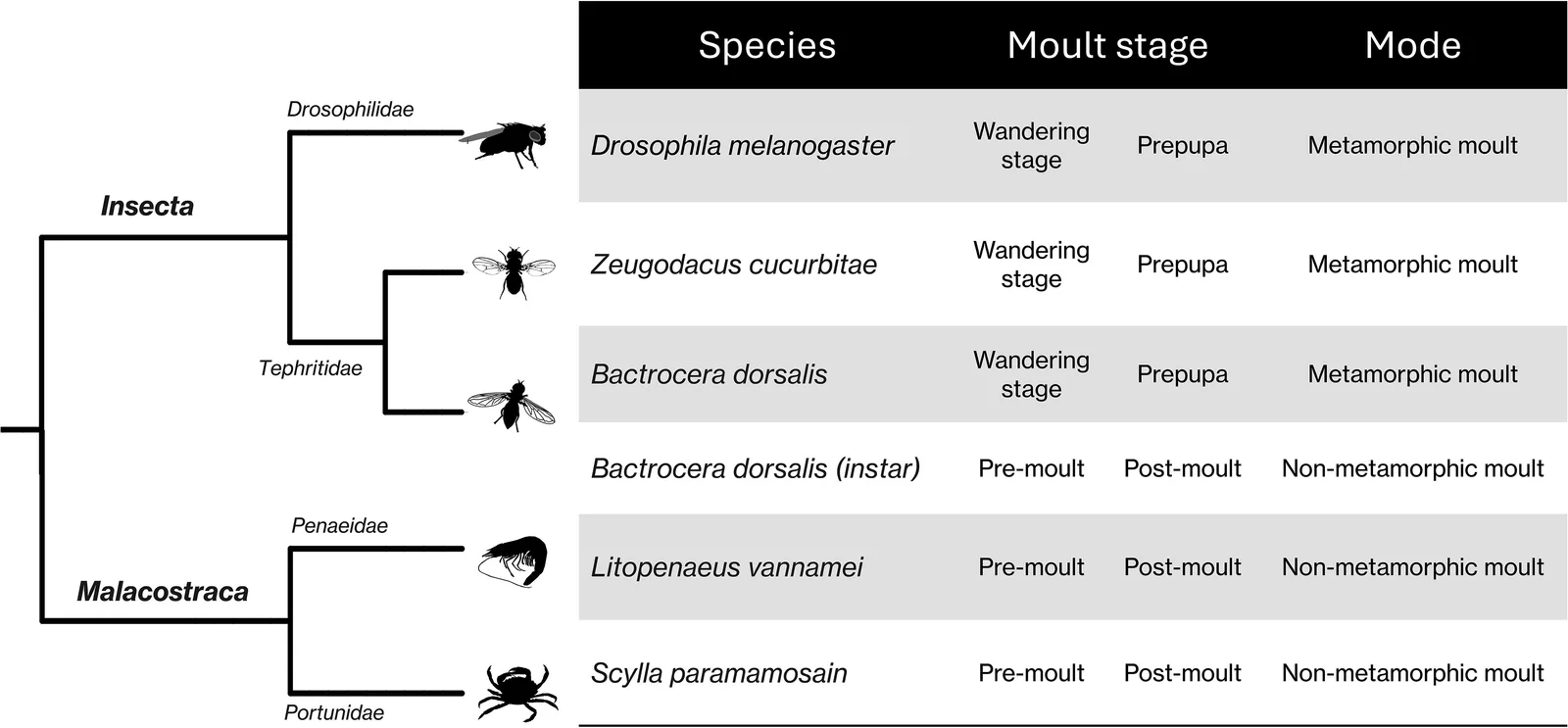
Phylogenetic tree showing the species used in this study. Additional information for each sample used is shown in the table on the right. Icon silhouettes under a public domain license were retrieved from Phylopic.

In this study, we investigated gene expression conservation of molting using publicly available transcriptomic datasets across several insect and malacostracan species, the two largest monophyletic classes of pancrustaceans. We took two approaches to this question. First, in vertical comparison, we examined the gene expression of ecdysis sensu stricto and pupariation in flies (larval–prepupal molt) across five representative pancrustacean species (Table 1). Although the terminology for these processes varies in the literature, we specifically refer to molting modes in this study as either metamorphic, such as larval–prepupal molt in insects, or nonmetamorphic, i.e. the instar–instar molt. Of note, specific stages are not necessarily directly homologous across pancrustaceans. We examine shared gene expression patterns, and thus biological processes, during molting in different species and life stages. While larval–prepupal transitions in insects are often viewed through the lens of metamorphosis, larval–prepupal molts in insects are also hormonally driven molting events characterized by a surge in 20-hydroxyecdysone (20E), a period of quiescence (nonfeeding phase), cuticle synthesis and involve similar processes to postecdysial modifications such as cuticle sclerotization and tanning (White and Ewer 2014; Roer et al. 2015). Despite the well-established role of 20E in triggering both molting and larval–prepupal molt, downstream pathways and developmental programs that are potentially conserved between these two processes beyond insects remain largely unexplored. Secondly, in horizontal comparison, we examined gene expression conservation across several timepoints of molting for *D. melanogaster* and *Litopenaeus vannamei*, the only species in our dataset with time-series data (Table S1). This allowed us to examine how gene expression dynamics shift across successive timepoints, and whether expression conservation patterns are consistent across the entire molting process. We found that gene expression of molting is characterized by both highly conserved and lineage-specific signatures, and that this pattern is not uniformly distributed. The mid-phase of the molting is more lineage-specific, exhibiting an inverse hourglass pattern. Our findings highlight the evolutionary conservation of molting and provide insights into the evolution of molting strategies in Pancrustacea (Fig. 1).

**Table 1:** Summary of datasets used for gene expression analysis

Taxonomic group	Species	Molt stages	Source
Insecta	*Drosophila melanogaster*	Wandering stage	(Graveley et al. 2011)
		Prepupa	
	*Zeugodacus cucurbitae*	Wandering stage	(Wei et al. 2020; Ward et al. 2021)
		Prepupa	
	*Bactrocera dorsalis*	Wandering stage	(Liu et al. 2020)
		Prepupa	
		Instar premolt	
		Instar postmolt	
Malacostraca	*Litopenaeus vannamei*	Premolt	(Gao et al. 2015)
		Postmolt	
	*Scylla paramamosain*	Premolt	(Liu et al. 2022)
		Postmolt	

## Results

### Overall Gene Expression Patterns Cluster by Lineage and Molt Stages

**Fig. 2.**
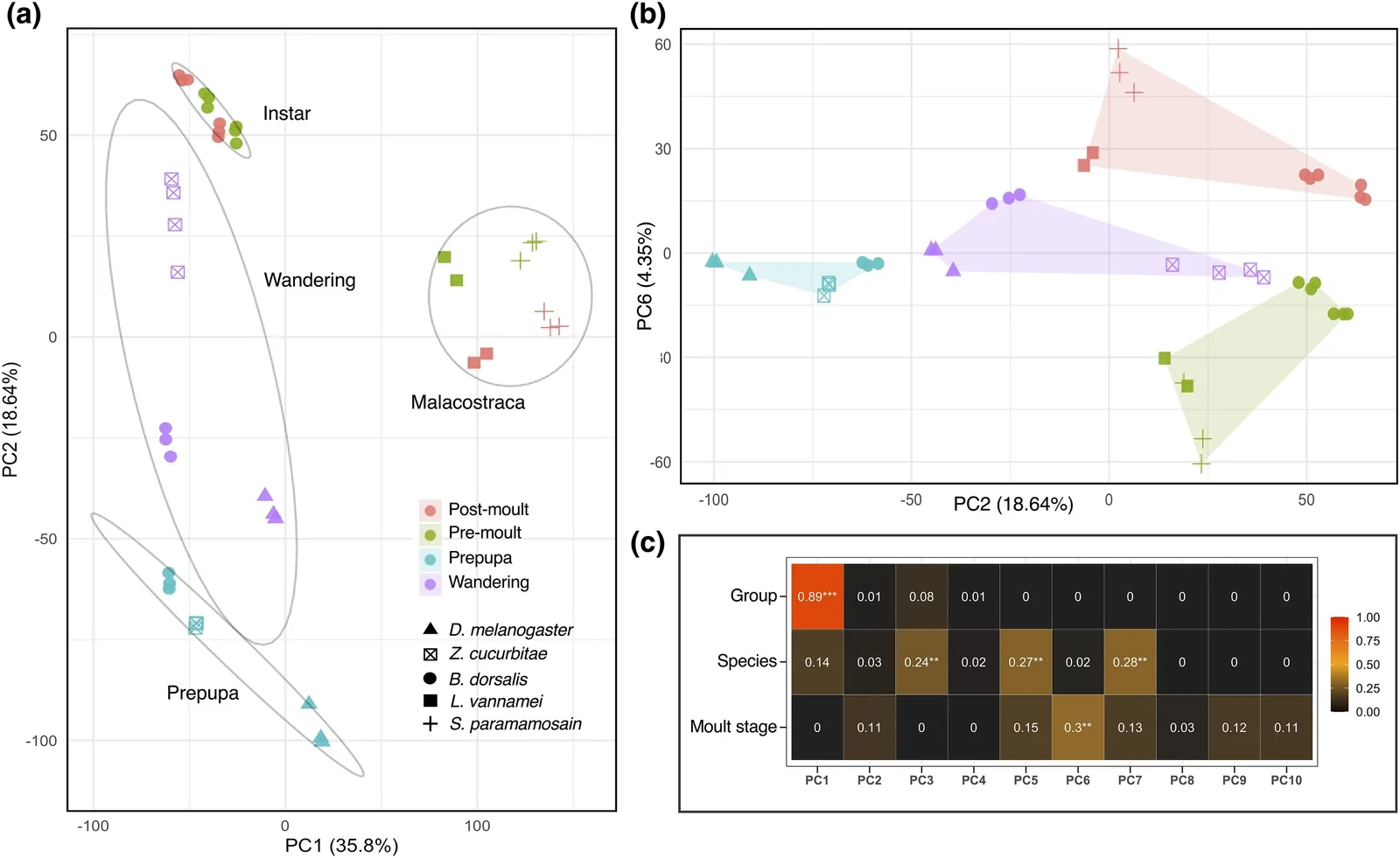
Principal component analysis of RNA-Seq data. a) PC1 and PC2 cluster samples by taxonomic group and molt stage. b) PC2 and PC6 show clustering of samples by molt stage. Left and top portions show prepupa and postmolt stage, while the samples at the center and bottom show wandering and premolt stages. c) Eigenvalue correlations of the principal components with respect to taxonomic group, species, and molt stage. Color indicates the strength of the correlation, and asterisks indicate the level of significance after Benjamini–Hochberg (Benjamini and Hochberg 1995) correction with *P < 0.05, **P < 0.01, and ***P < 0.001.

To examine the gene expression pattern of molting dataset across all samples, we performed a principal component analysis (PCA) on the VST RNA-seq counts for the 5,543 single-copy orthogroups identified across all species (Fig. 2). The PCA shows both clear molting stage clustering consistent with the PCA done on individual species datasets (Fig. S6) and strong separation between insect and malacostracan lineages, indicating that lineage-specific variation represents an additional major axis of biological variation. These results indicate that the dominant patterns observed in the dataset primarily reflect biological rather than technical variation. The first 10 principal components (PCs) explained 95% of the variance in gene expression, with PC1 and PC2 alone accounting for 54%. We tested whether these PCs were significantly associated with either molt stage, subphylum, or species (Fig. 2c). The strongest correlation with molt stage was PC6 and plotting PC2 and PC6 clustered samples by molt stages. Among the genes that contribute the most to PC 6 are genes known to be involved in molting, such as hormone receptor 3 (*Hr3*), ecdysone induces protein 78c (*Eip78c*), ecdysis triggering hormone receptor (*ETHR*), and various cuticular proteins and chitinases (Table S2). There are also uncharacterized genes, such as CG11892 which has predicted ecdysteroid-22 kinase activity, and CG12009, which has a predicted chitin-binding peritrophin-A domain. All samples were derived from whole-body extracts, yet despite variable involvement of different tissues in molting, we detected a clear signal of molting in the dataset. This likely reflects the large changes in gene expression just before and after ecdysis and pupariation.

### Shared and Lineage-specific Differentially Expressed Genes in Nonmetamorphic Molts

**Fig. 3.**
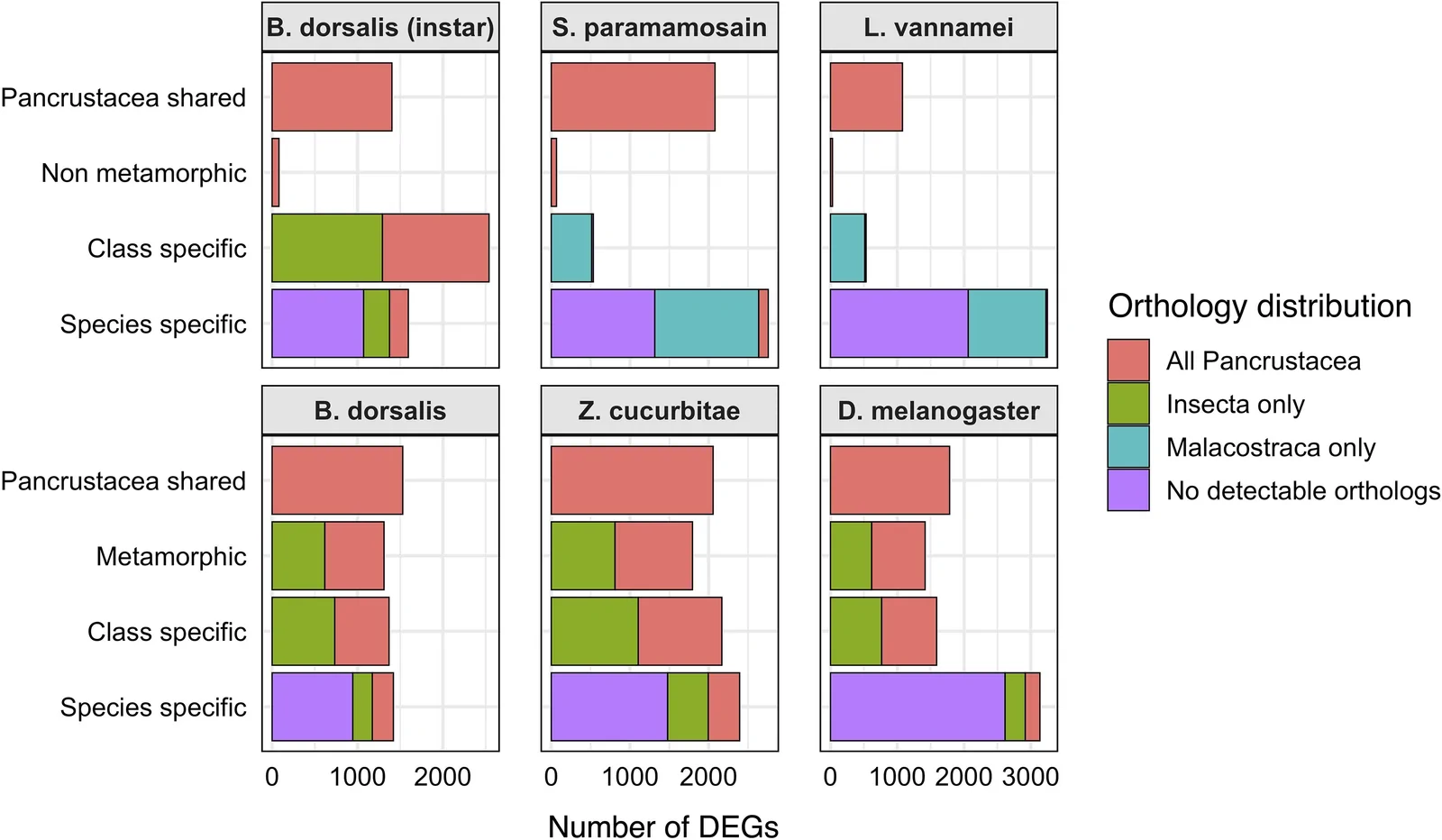
Classification of differentially expressed genes (DEGs) and their corresponding orthology distribution. Top: nonmetamorphic molts; bottom: metamorphic molts. “Class specific” are DEGs restricted to Malacostraca or to insects.

We first consider only the nonmetamorphic molts, i.e. the instar molt of Bactrocera *dorsalis* and the two malacostracans (Fig. 3, top). While the largest fraction of differentially expressed genes (DEGs) between pre- and post-molt are classified as lineage-specific (i.e. species-specific or class-specific), there are also many DEGs which are shared among pancrustaceans.

These shared DEGs include known members of the late genes and ecdysis neuromotor pathways, such as *knickkopf* (*Knk*), *obstructor-A* (*Obs-A*), retroactive *(Rtv), serpentine* (*serp*), *krotzkopf verkehrt* (*kkv*), and various chitin deacetylases and chitinases (Moussian et al. 2015; Pesch et al. 2015; Pesch et al. 2016; Campli et al. 2024). In addition, many of these DEGs are involved in cuticle development and include various esterases, ecdysteroid kinases, and structural components such as extracellular matrix proteins, gap junction elements, and other support structures (Table S3). We also found cytochrome P450 (CYP) genes and other steroid binding proteins and transporters, some of which have been shown to be involved in ecdysone metabolism and sterol transport, such as organic anion transporter polypeptides, Niemann-Pick type C (*NPC*), δ-aminolevulinic acid synthase (*Alas*), megalin, and membrane steroid binding protein (*MSBP*) (Huang et al. 2005; Fujii-Taira et al. 2009; Riedel et al. 2011; Nakaoka et al. 2017). Many of these genes have orthologues outside Pancrustacea, indicating that their involvement in molting might be conserved more broadly.

### Comparison of Metamorphic and Nonmetamorphic Molts

To examine whether these Pancrustacea-shared DEGs are restricted to nonmetamorphic molts or are conserved more broadly across molting modes, we extended the comparison to include metamorphic molts in insects (Fig. 3). Interestingly, only a small fraction of DEGs was exclusively shared among nonmetamorphic molts, whereas Pancrustacea-shared DEGs were broadly represented among DEGs in metamorphic molts. This indicates that many conserved components of the molting program are deployed in both molting modes.

There were differences between insects and malacostracans in the number of species-specific compared to class-specific DEGs. Malacostracan species exhibited a higher proportion of species-specific DEGs than insects; splitting them between upregulated and downregulated genes shows similar trends (Fig. S1). This pattern likely reflects the greater divergence among species within each group. *L. vannamei* and *S. paramamosain* belong to distinct decapod groups Dendrobranchiata and Pleocyemata (Reptants), respectively, which diverged approximately 400 million years ago. They exhibit markedly different life histories and ecology, with *L. vannamei* fully aquatic and *S. paramamosain* semi-terrestrial. In contrast, *B. dorsalis*, *Z*. *cucurbitae* (both Tephritidae), and *D*. *melanogaster* (Drosophilidae) diverged only around 150 million years ago (estimated using TimeTree: Kumar et al. 2022).

In addition to differences in DEG numbers, class-specific DEGs differed markedly in their orthology distribution between insects and malacostracans (Fig. 3). In the two malacostracans, class-specific DEGs were predominantly composed of Malacostraca-restricted orthologous groups. In contrast, class-specific DEGs in insects included a substantial fraction of Pancrustacea-shared orthologous groups. Across all species, species-specific DEGs were strongly enriched in genes with lineage-restricted orthogroups and in genes with no detectable orthologs, indicating that transcriptional novelty during ecdysis is primarily driven by genes with lineage-restricted orthology distribution rather than by changes in expression of conserved orthologous groups.

### Gene co-expression Analysis Shows Conserved Gene Expression Pattern of Molting

**Fig. 4.**
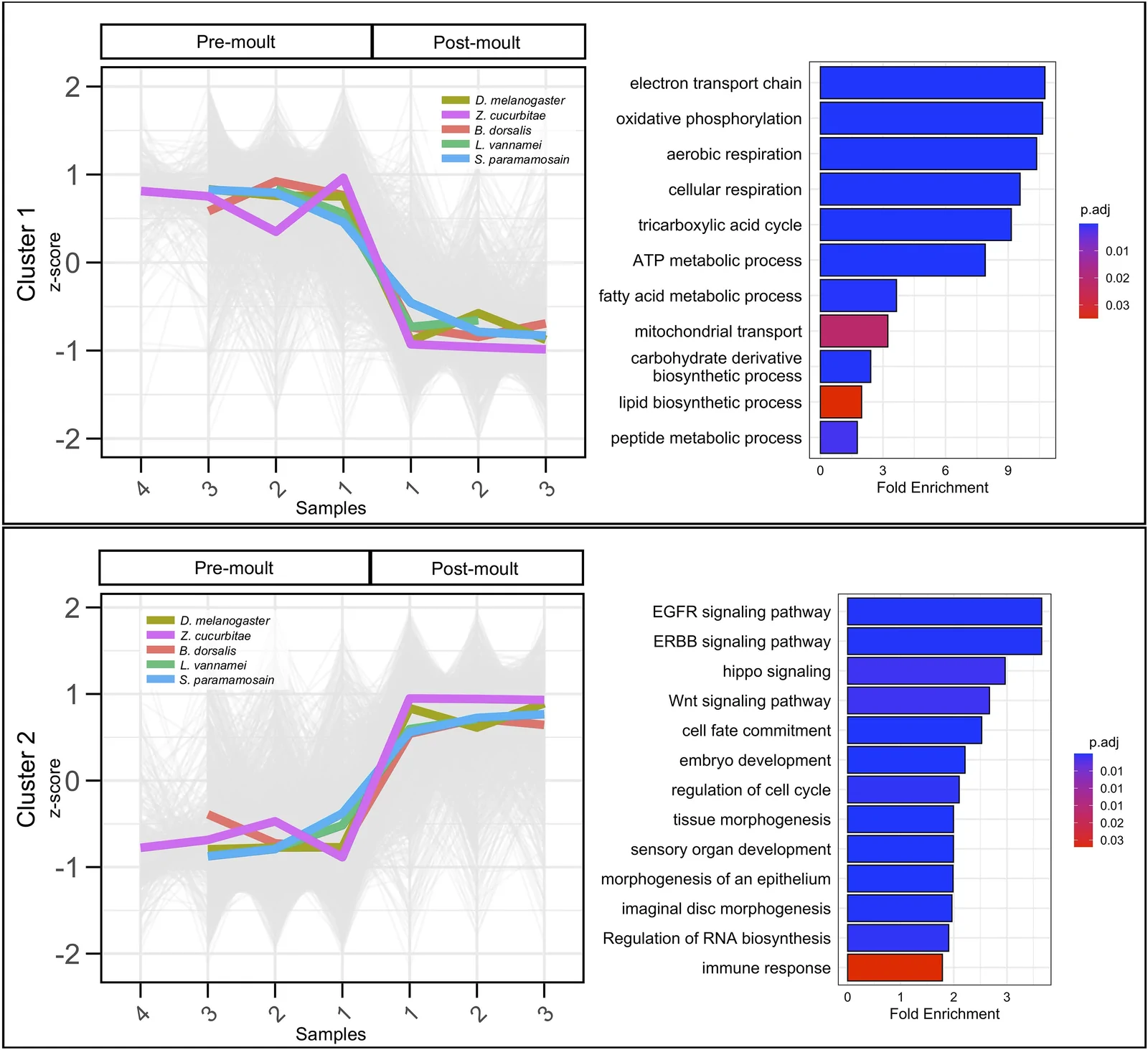
Clusters of co-expressed genes in molting. The strength of expression in z-scores (*Y*-axis) is illustrated for the different molt stage replicates per species, where colored lines represent the expression mean while gray lines represent individual gene expression values per replicate (*X*-axis). Gene ontology (GO) enrichment plots showing representative significant terms for each cluster are shown on the right side.

**Fig. 5.**
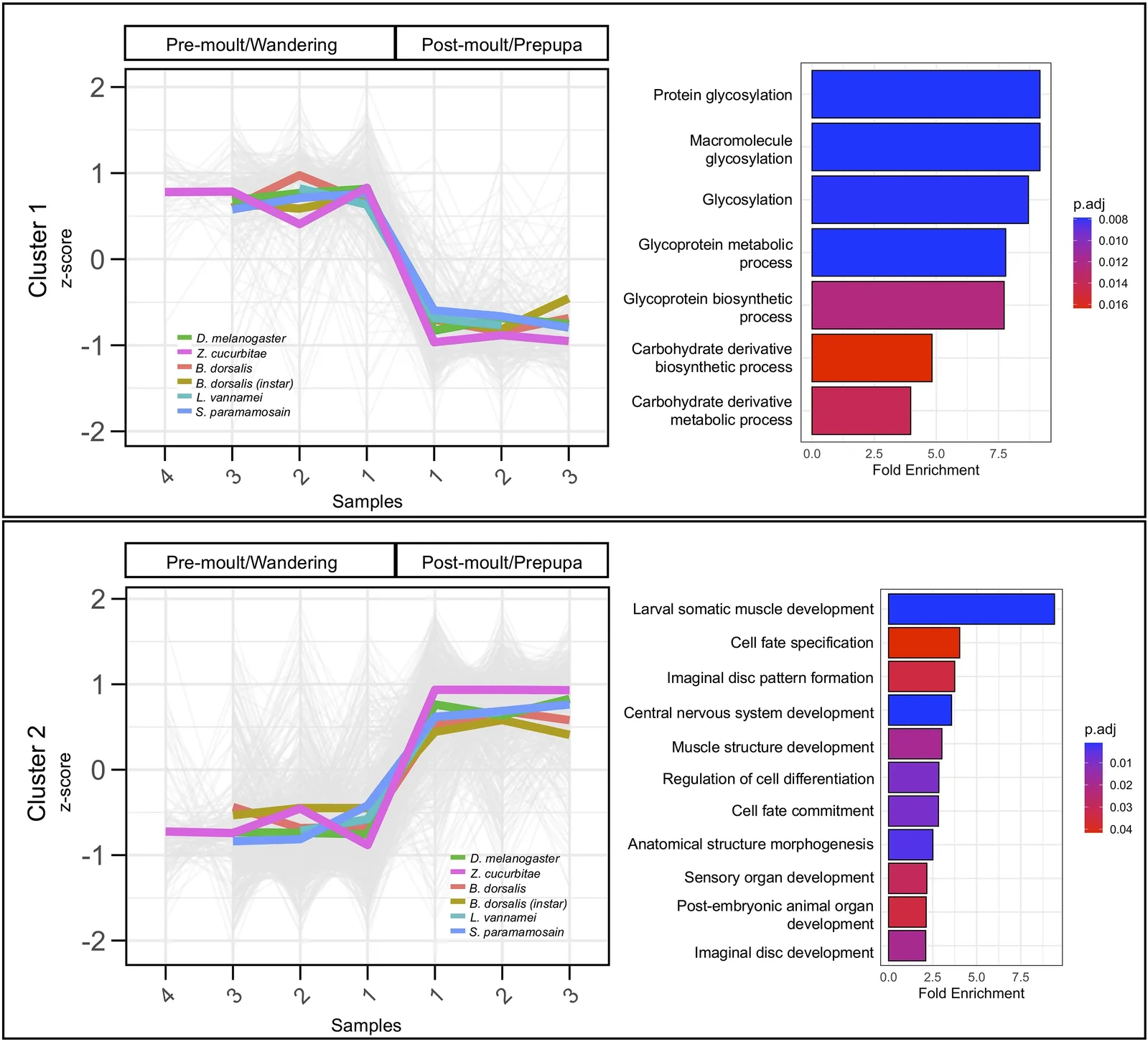
Clusters of coexpressed genes in molting (with *B. dorsalis* instar). The strength of expression in z-scores (*Y*-axis) is illustrated for the different molt stage replicates per species where colored lines represent the expression mean while gray lines represent individual gene expression values per replicate (*X*-axis). Gene ontology (GO) enrichment plots showing representative significant terms for each cluster are shown on the right side.

Most genes function within biological pathways or coexpression networks, and concerted expression changes of distinct gene sets can provide valuable insights into various coregulated gene modules involved in molting. We performed a coexpression analysis of all 5,543 orthologues across all species and identified gene clusters with similar expression patterns (Fig. 4). We recovered two major clusters that exhibit consistent expression across all samples: cluster 1 consists of genes that are downregulated during the postmolt stage (Fig. 4: top panel), and cluster 2 consists of genes that are upregulated during the postmolt stage (Fig. 4: bottom panel). A total of 774 coexpressed genes (14% of all orthologues) were identified across the five species (Table S4). When the instar molt sample from *B*. *dorsalis* was included in the analysis, the number of coexpressed genes decreased to 184 genes (3% of all orthologues) (Fig. 5).

Functional analysis of the 774 coexpressed genes shows that genes in cluster 1 are enriched in terms related to core metabolic and cellular processes, such as cellular respiration and energy metabolism. Meanwhile, cluster 2 is enriched for terms related to tissue morphogenesis and developmental signaling, suggesting that the postmolt stage is characterized by the activation of pathways associated with cell proliferation, differentiation, and tissue remodeling, as expected. In addition to this, we also recovered immune-related genes in cluster 2, including those known to mediate melanotic encapsulation of foreign targets, such as *zizimin-related* (*zir*), *ephexin* (*exn*), *Rho guanine nucleotide exchange factor 3* (*RhoGEF3*), and *Rho-related BTB domain-containing protein* (*RhoBTB*), and Toll and IMD signaling pathway genes including *Relish*, *supernumerary limbs* (*slmb*), *activating transcription factor-2* (*ATF-2*), *spatzle 3* (*spz3*), and *caspase* (*casp*).

The coexpressed gene sets which include the *B. dorsalis* instar-stage are not enriched for genes associated with cellular respiration or signaling pathways (Fig. 5). Instead, for cluster 1, the enriched Gene Ontology terms were limited to processes such as protein glycosylation and carbohydrate metabolism. For cluster 2, we continued to recover terms related to tissue morphogenesis, sensory organ development, and muscle development. However, terms associated with key signaling pathways such as *Wnt*, *Hippo*, and *EGFR* were no longer recovered. This suggests that the metabolic and developmental requirements of instar molt in *B. dorsalis* might be different from other samples included in this analysis.

### Molting Gene Expression Exhibits Varying Patterns of Conservation

**Fig. 6.**
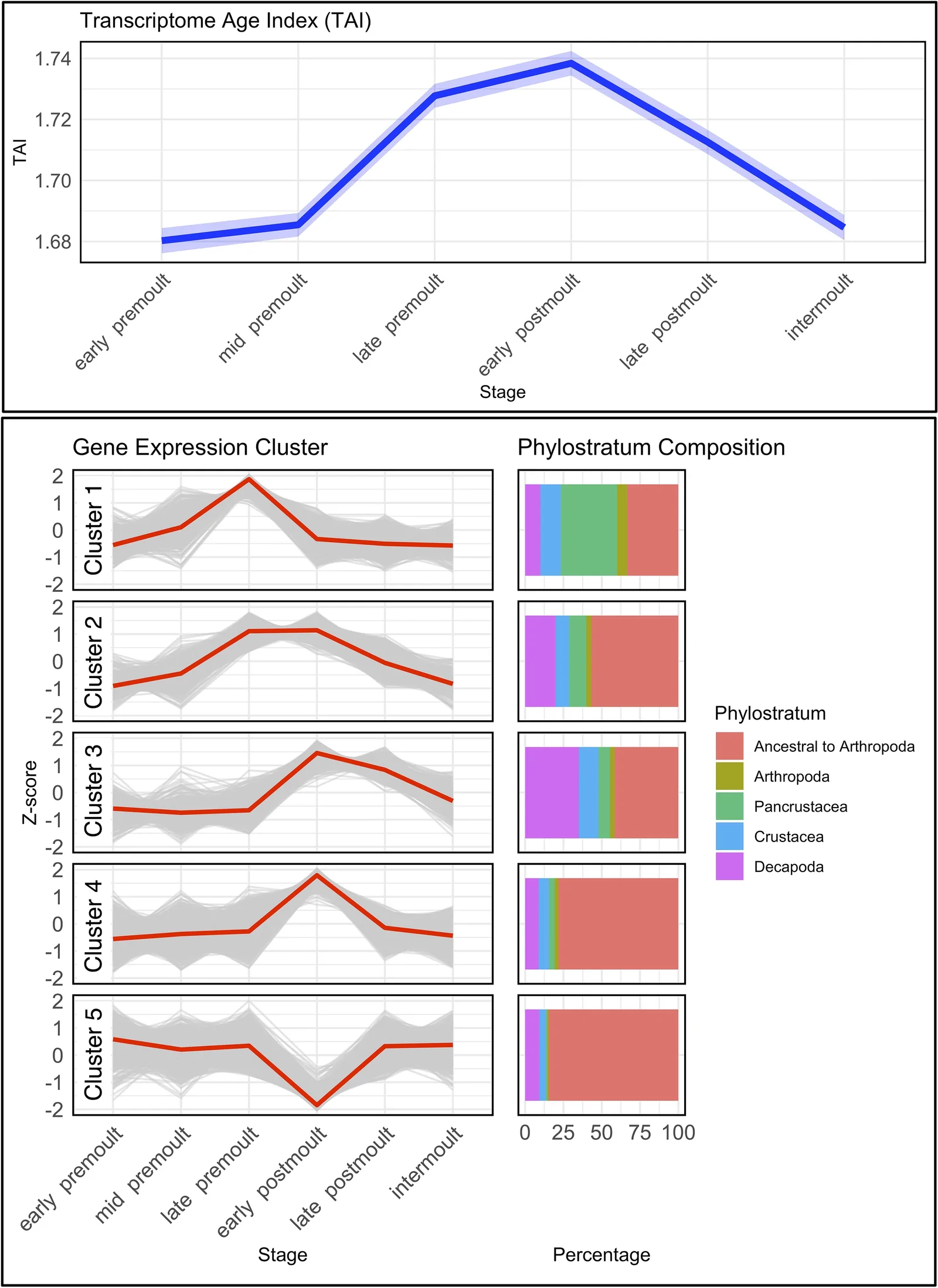
Transcriptome age index (TAI) and molting gene expression clusters in *L. vannamei*. Top panel shows the TAI of various molt stages in *L. vannamei* (*P* < 0.0001). Shaded line area indicates ±1 standard deviation. Bottom panel shows the gene expression clusters of *L. vannamei* molt samples and their corresponding phylostratum composition.

**Fig. 7.**
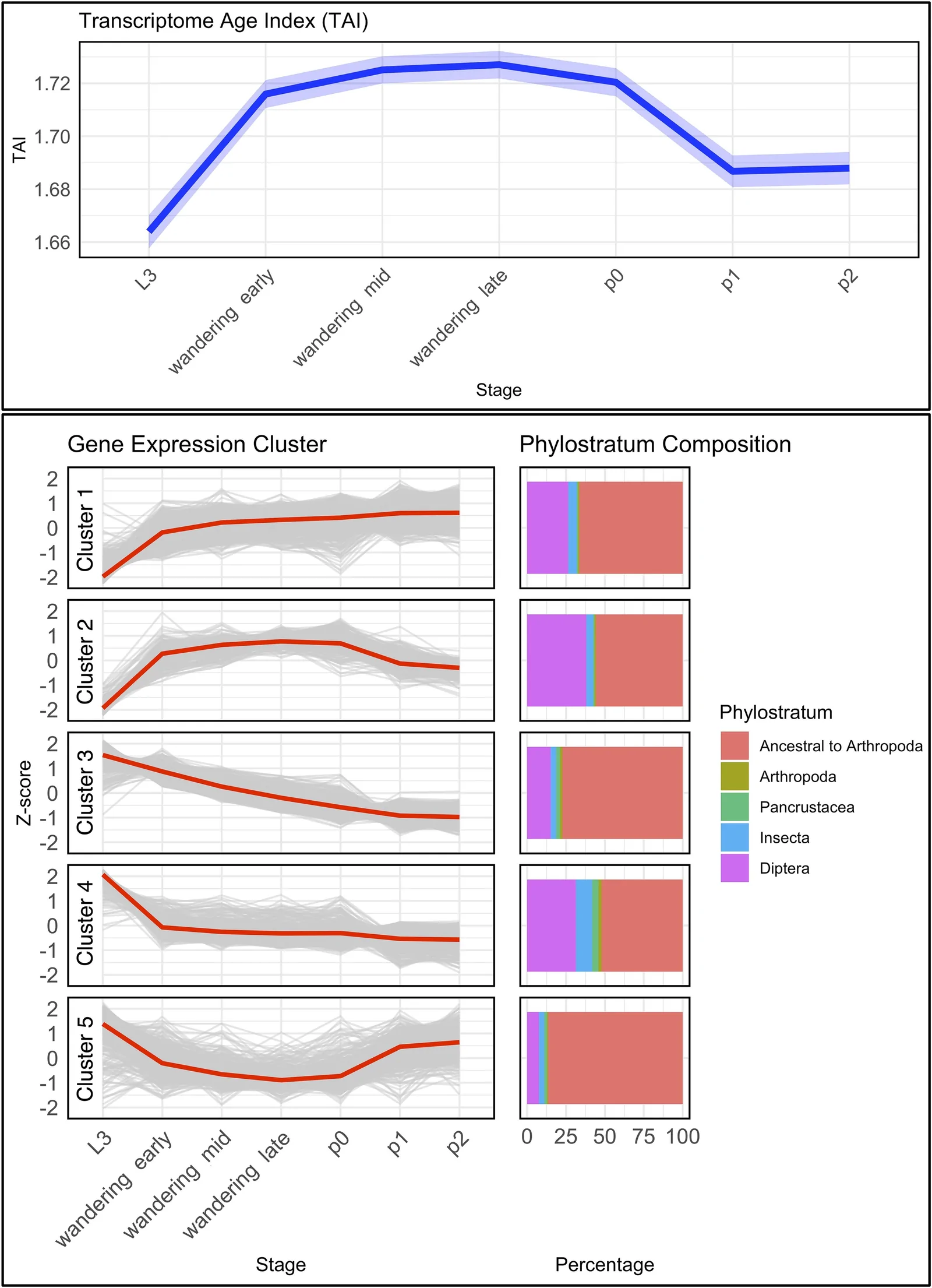
**Transcriptome age index (TAI) and molting gene expression clusters in *D. melanogaster***. Top panel shows the TAI of various molt stages in *D. melanogaster* (*P* < 0.0001). Shaded line area indicates ±1 standard deviation. Bottom panel shows the representative gene expression clusters of *D. melanogaster* samples and their corresponding phylostratum composition.

Despite recovering a subset of genes that are coexpressed across all species, most genes did not show consistent coexpression patterns across species and groups. This suggests that molting gene expression is for a large part lineage-specific, with gene expression dynamics varying between species. However, molting as a process involves more than just the two timepoints around ecdysis. Thus, we expanded our comparison to include additional timepoints from pupariation in *D. melanogaster* and molting in *L. vannamei*. We then examined transcriptome age patterns (TAI) across these timepoints. We found that conservation of gene expression is not evenly distributed across molting in *L. vannamei* and larval–prepupal molt in *D. melanogaster*. For both species, TAI during the middle stages tends to be younger (i.e. more lineage-specific) showing an inverse hourglass pattern (Figs. 6 and 7). However, it should be noted that while transcriptome age index (TAI) analysis is a widely used method to assess the evolutionary age of gene expression profiles across developmental stages, it is also known to be sensitive to certain biases (Piasecka et al. 2013). One of the main challenges is that TAI relies on the mean expression values of genes, which can be skewed by a small number of very highly expressed genes such as housekeeping genes. These genes often have stable and abundant expression across conditions and may mask the contribution of biologically meaningful signals from genes with lower expression. As previously shown, removing or correcting for the influence of highly expressed genes can substantially alter the resulting TAI patterns, often revealing an additional layer of conservation pattern that were otherwise hidden (Piasecka et al. 2013), although this is moderated by the use of transformed expression values (Liu and Robinson-Rechavi 2018). Moreover, the differences in TAI that we observe between stages are very small relative to the range of possible values. To assess whether the observed TAI patterns were driven by highly expressed but invariant genes, we repeated the analysis after filtering genes based on expression variance across stages. Specifically, we removed the lowest 10%, 25%, and 50% of genes ranked by variance and recomputed TAI. In all cases, the overall pattern was preserved, indicating that dynamically expressed genes contribute most strongly to the enrichment of younger genes during the middle stages of molting (Fig. S7). These results suggest that the observed enrichment of evolutionarily younger genes is not simply an artifact of constitutive expression but is associated with the most dynamically expressed components of the molting transcriptome. To further address this limitation, we also performed a temporal coexpression analysis to identify clusters of genes with similar expression patterns.

We identified clusters of genes showing distinct trends across the molting process (Figs. 6 and 7). For each of these clusters, we recovered varying patterns of conservation based on their Phylostratum composition. In *L. vannamei*, most of the gene clusters were consistent with TAI analysis. Clusters with higher expression in the middle of the molt process tend to be composed of younger genes, while those with lower expression during this phase are enriched for older, more conserved genes. For example, cluster 1, which shows peak expression in the late premolt stage, has a higher proportion of genes assigned to phylostrata dating back to the Pancrustacea node. In contrast, the relative contribution of more recent nodes, particularly those specific to Decapoda, increases toward the postmolt stages. On the other hand, cluster 4, which peaks in early postmolt, deviates from the overall pattern by being composed largely of older genes, suggesting a possible reactivation of conserved programs following ecdysis. Although most gene clusters are dominated by younger phylostrata, especially during the middle of the molt cycle, it is worth noting the changes of phylostrata coming from Pancrustacea and Decapoda. This shift in phylostratum composition from a greater contribution of Pancrustacea-level genes in the late premolt stage to increased representation of Decapoda-level genes in the postmolt stage reflects the stage-specific recruitment of conserved and lineage-specific processes across the molting process.

Younger gene clusters were enriched in terms involved in immune response, nervous system formation, sensory organ development, peroxidase activity, pigment binding, and cuticle development (Fig. S2). Cuticle development is enriched in clusters 1 to 3, especially in cluster 1. We confirmed this with cuticleDB (Magkrioti et al. 2004; Ioannidou et al. 2014) (Fig. S3). Genes that are predominantly expressed in cluster 3 (postmolt) are enriched in terms related to carbonate dehydratase and pigment binding, which implies the stage-specific expression of genes potentially involved in calcification and pigmentation. Clusters with older phylostratum composition were enriched in terms related to extracellular matrix organization, fatty acid binding, and conserved metabolic processes such as steroid and carbohydrate metabolism, proteolysis, and lipid biosynthesis. These functional interpretations are based on Gene Ontology (GO) annotations, which can be broad and may reflect pleiotropic functions. We therefore interpret these enrichments as putative functions derived from computational annotation that require further experimental validation.

In *D. melanogaster*, we recovered 10 gene expression clusters with varying temporal coexpression patterns (Fig. 7; Fig. S4). The gene clusters that we recovered are largely not consistent with the TAI analysis, with mixed patterns (Fig. 7). Most of the clusters exhibit a notable contribution of Diptera-specific genes, which is consistent with previous observations on the recruitment of younger genes in life stage transitions of flies (Remmel et al. 2025). Most clusters were dominated by older phylostrata and enriched in cellular and metabolic processes (Fig. S5). Cluster 1 is enriched in terms related to cilium movement and sperm flagellum assembly; cluster 2 is enriched in gamete development such as microtubule polymerization, spermatogenesis, and spermatid differentiation, while cluster 3 is enriched in RNA biogenesis, body morphogenesis, and cuticle development.

## Discussion

Molting is a highly conserved developmental process across Arthropoda. While a core genetic toolkit for molting has been identified (Campli et al. 2024; Campli et al. 2026), largely derived from insect model systems, comparative genomic studies suggest that this “moulting genetic backbone” has undergone lineage-specific elaborations through gene gains and losses (Campli et al. 2026). The extent to which genes involved in molting beyond this core toolkit are conserved or lineage-specific remains poorly known. In this study, we analyzed gene expression datasets from insects and malacostracans to investigate the conservation of molting gene expression in Pancrustacea.

We identified DEGs that are conserved between distant Pancrustacea species including genes involved in ecdysteroid metabolism. In addition, we recovered genes involved in cuticle development and in postecdysial processes. They notably include genes involved in the structural organization of the cuticle. For example, *Knk*, regulated by *Rtv*, organizes chitin into laminae and protects it from chitinase-mediated degradation, along with chitin deacetylases known to direct proper cuticle organization (Chaudhari et al. 2011; Chaudhari et al. 2013; Moussian 2013). In the extracellular matrix, *Obst-A* binds the nascent chitin fibers produced by *Kkv* and recruits the *Serp/Knk* complex to the apical plasma membrane for chitin organization (Ostrowski et al. 2002; Petkau et al. 2012; Moussian 2013; Pesch et al. 2015). This complex is essential for aligning the chitin into an ordered structure and for protecting the developing matrix from premature degradation by chitinases. Loss of these genes results in molting defects, chitin disorganization, and lethality (Ostrowski et al. 2002; Chaudhari et al. 2011, 2013; Petkau et al. 2012; Moussian 2013; Pesch et al. 2015).

In addition to structural organization and chitin synthesis machinery, we also found genes involved in protein trafficking and membrane transport that are essential for cuticle turnover. The enrichment of genes related to lysosomal activity, protein trafficking, and endocytosis during ecdysis is likely due to chitin synthesis and degradation. As the old cuticle is degraded, endocytosis enables the uptake of cuticle fragments and extracellular debris, which are processed in lysosomes to recover reusable monomers, while the secretory pathways direct newly synthesized cuticular proteins, chitin synthases, and other structural components to the apical surface for assembly of the new cuticle (Moussian et al. 2007). Consistent with this, we identified core secretion pathway genes such as *ghost* (*Gho*), *Haunted* (*Hau/Sec23*), *Sec24*, and other COAT proteins, which are known to be essential for apical plasma membrane remodeling during cuticle differentiation (Moussian et al. 2007; Norum et al. 2010). The multiligand endocytic receptor *Megalin* (*Mgl*) in *Drosophila* illustrates another trafficking-dependent mechanism, where *Mgl*-mediated endocytosis of Yellow protein restricts pigment deposition to the distal procuticle, ensuring proper spatial organization of cuticle components (Christensen and Willnow 1999; Moestrup and Verroust 2001; Riedel et al. 2011). In insects, similar roles for endocytosis have been reported. In *Locusta migratoria*, clathrin-mediated endocytosis supports resource recycling during cuticle degradation, and its disruption causes molting defects, consistent with findings in *Helicoverpa armigera* where a G protein-coupled receptor mediates 20-hydroxyecdysone uptake via clathrin-dependent endocytosis (Kang et al. 2021; Shi et al. 2022). Evidence from *Caenorhabditis elegans* shows that disruption of endocytic trafficking in epidermal cells leads to molting arrest, defective clearance of the old cuticle, failure to internalize cargoes such as steroid hormone precursors, and disruption of plasma membrane homeostasis during new cuticle secretion (Yochem et al. 2015; Lažetić and Fay 2017). The occurrence of such trafficking-dependent mechanisms in nematodes suggests that they represent an ancestral molting machinery that predates Arthropoda.

Genes coexpressed during ecdysis are not limited to those directly involved in cuticle synthesis and organization. Instead, molting also recruits evolutionarily conserved pathways supporting broader physiological functions, including metabolism, immune response, cellular differentiation, and tissue morphogenesis. Molting is an energy-intensive process such that the caloric loss associated with shedding the exuviae is substantial (Harvey et al. 1980; Sweeney and Vannote 1981). Additionally, insects cease feeding behaviors before, during, and after the moult (Ayres and MacLean 1987; Stamp and Bowers 1994; Rackauskas et al. 2006; Kang et al. 2019). Similarly, in decapod crustaceans, feeding also declines during the premolt phase, stops entirely during ecdysis, and resumes only in the late postmolt period (Dall 1986; Harpaz et al. 1987; Chan et al. 1988). This feeding behavior reflects the mechanical and physiological challenges posed by exoskeleton shedding, particularly affecting essential feeding structures such as the buccal cavity, esophagus, and anterior stomach (Lemos and Weissman 2021). Previous studies indicate that ATP production is regulated by 20E signaling (Chang et al. 2022) and premolt is characterized by high energy metabolism which has been supported both at transcriptomic and proteomic levels (Head et al. 2019). In various decapod crustaceans, the duration of premolt to postmolt can take a substantial amount of time during its molt cycle (Drach and Tchernigovtzeff 1967; Lemos and Weissman 2021; Gorissen and Sandeman 2022; Kim et al. 2024), and the expression of these genes ensures sufficient energy supply during ecdysis.

We also identified genes involved in the melanization pathway, which plays a key role in cuticle hardening during the postecdysial period (Hiruma and Riddiford 2009). Moreover, we recovered immune-related genes, including components of the Toll and IMD pathways. The melanization pathway itself occupies an interesting intersection between cuticle maturation and innate immunity, functioning both in cuticle hardening and in pathogen defence (Marmaras et al. 1996; Vavricka et al. 2010; Beckage 2011; Nakhleh et al. 2017). The vulnerability of arthropods to infection is particularly heightened after ecdysis, when the protective cuticle is incompletely formed. Whether the coexpression of immune genes alongside cuticle maturation reflects the vulnerability that accompanies molting remains unclear, but previous studies suggest that immune activation may provide protective benefits during this period (Tang 2009; Clark and Strand 2013; Zhang et al. 2014; Louradour et al. 2017). The melanization pathway is triggered by PAMPs, such as bacterial peptidoglycan or fungal β-glucans. This activates a protease cascade that converts proPO to active PO, which oxidizes phenols into quinones. These reactive intermediates polymerize to form melanin, which is deposited at sites of infection to encapsulate pathogens and generate cytotoxic reactive oxygen species (Vavricka et al. 2010; Beckage 2011; Nakhleh et al. 2017; Shan et al. 2023). Melanization has been extensively studied in insects such as *D. melanogaster* and *B. mori*, where it enhances survival during pathogenic infection (Zhang et al. 2014; Louradour et al. 2017), interacts with hemolymph coagulation (Bidla et al. 2005), and contributes to broader immune defence (Tang 2009; Clark and Strand 2013). Similar immune-related responses during molting have been observed in decapod crustaceans. In *S. paramamosain*, immune-related genes involved in antimicrobial peptide synthesis, Toll signaling, phenol oxidase system, and antioxidant pathways are significantly upregulated in the hepatopancreas during postmolt and intermolt stages (Xu et al. 2020). Ortholog coexpression of these gene sets suggests that they play a conserved role during ecdysis.

The coexpression patterns that we observe differ significantly depending on life history strategy. When instar molt stages from holometabolous insects are included, we recover markedly fewer coexpressed genes, particularly those involved in signaling and differentiation pathways. The Hippo and Wnt signaling pathways are important regulators of tissue homeostasis and organ size that coordinate cell proliferation, differentiation, and apoptosis (Wodarz and Nusse 1998; Komiya and Habas 2008; Halder and Johnson 2011). In holometabolous insects, growth and differentiation are decoupled (Aldaz and Escudero 2010; R. F. Chapman et al. 2013; Manthey et al. 2024). During larval development, certain populations of cells, the imaginal primordia, remain in an undifferentiated, pluripotent state with latent embryonic potential and resume differentiation during pupal transition (Aldaz and Escudero 2010; R. F. Chapman et al. 2013). In holometabolous insects, molting during the instar stages likely supports mostly growth and cuticle replacement. Extensive studies over the past decades have established that the transition between molts that are dedicated to growth and the extensive differentiation involved in metamorphosis is regulated by a complex interplay of endogenous and environmental factors, involving hormonal feedback between ecdysteroids, *juvenile hormone* (*JH*), and stage-specifying transcription factors such as *Chinmo, Krüppel-homolog 1* (*Kr-h1*), *Broad*, and *E93* (Truman et al. 2006; Suzuki et al. 2013; Truman 2019; Truman and Riddiford 2019, 2022). Gradual elaboration of this hormonal crosstalk might have led to the evolution of metamorphosis, by coupling molt cycles to environmental and physiological signals that determine whether an individual proceeds to another juvenile stage or transitions to the adult form. Our results suggest that the molting program can be decoupled from broader developmental processes depending on life history strategy.

Surprisingly, the coexpressed genes associated with signaling and differentiation pathways were shared primarily between crustacean molts and insect metamorphic molts rather than with instar larval molts. In insects, EGFR signaling has been implicated in regulating both ecdysteroid and JH biosynthesis during developmental transitions (Cruz et al. 2020; Li et al. 2022). Likewise, components of EGFR/ErbB and MAPK signaling are strongly represented in the crustacean Y-organ (Mykles 2021). Pathways such as EGFR/MAPK signaling might represent a conserved mechanism involved in hormonal regulation of molting. Although tissue remodeling during crustacean molting is less extensive than in holometabolous metamorphosis, each molt nevertheless involves coordinated processes of tissue growth and differentiation (Covi et al. 2010; Kim et al. 2024; Adachi et al. 2025). In addition, differentiation pathways such as Wnt signaling have been implicated in setal development in crustaceans (Wang et al. 2023), and setal structures themselves are regenerated during each molt cycle and form the basis of many molt staging systems in decapod crustaceans (Gorissen and Sandeman 2022; Kim et al. 2024). While the precise functional role of these differentiation pathways in hormonal regulation of molting in crustaceans remains to be tested, this suggests that metamorphic holometabolous molt might have derived from remodeling in pancrustacean nonmetamorphic molt.

Despite identifying a small set of genes which are coexpressed across all five species, most of the genes that are differentially expressed during molting are lineage-specific, either because they do not have orthologs outside of one lineage, or because the differential expression pattern is restricted to one lineage even though the orthologs are more broadly distributed (Fig. 3). Temporal coexpression patterns show that these lineage-specific signatures are not evenly distributed across the molting process. Mid-molting gene clusters are enriched for relatively younger genes. In *L. vannamei,* these clusters are enriched in functional terms involved in the development of sensory structures, nervous system formation, pigment binding, and chitin-based extracellular matrix components. In contrast, older gene clusters are enriched for functional terms such as steroid metabolic processes, chitin catabolism, and carbohydrate metabolism. This pattern suggests that mid-molt lineage-specific signatures are driven in part by the development of the cuticle and specialized structures of the exoskeleton.

The cuticular surface is remarkably diverse, featuring a wide array of pigmentation and ornamentation, as well as specialized structures (Hallberg and Skog 2010; Mellon 2012). This includes various setal structures which line the exoskeleton and are periodically developed with each molt (Drach and Tchernigovtzeff 1967; Gorissen and Sandeman 2022; Kim et al. 2024). Many of these are innervated by sensory neurons which may house mechanoreceptive and chemoreceptive neurons, or both (Shields et al. 2008; Hallberg and Skog 2010; Mellon 2012). Studies combining physiological analysis with scanning electron microscopy have shown that many cuticular setae on locomotory, feeding, and grooming appendages are bimodal in function, containing both chemo- and mechanoreceptor cells (Shields et al. 2008; Hallberg and Skog 2010). These structures often serve species-specific roles, including mating behavior, mechano-sensation, and predator detection. Their diversity and functional specificity across lineages likely explain the recruitment of younger, lineage-specific genes in their regeneration during molting.

Lineage-specific genes have been implicated in species-specific adaptations for behavior, environmental factors, reproduction, host pathogen interactions, and speciation (Khalturin et al. 2008, 2009; Han et al. 2024). While it is intuitive to conclude that lineage-specific signatures of molting are due to adaptive divergence, some studies have shown that lineage-specific genes are not always adaptive. Young, lineage-specific genes can be gained, lost, or integrated into gene regulatory networks through stochastic processes, and may persist without signatures of adaptation (Palmieri et al. 2014; Vakirlis et al. 2022; Wacholder et al. 2023). At the same time, the phylogenetic breadth of the species included in this study represents an important limitation. Future studies including additional crustacean lineages, particularly nonmalacostracans, as well as other molt types and insect groups such as hemimetabolous insects, will be necessary to determine whether similar lineage-specific gene expression patterns are broadly conserved across Arthropoda. These lineage-specific genes thus present interesting candidates for future tests of adaptive evolution in arthropods.

While the overall transcriptome age pattern is similar between *L*. *vannamei* and *D*. *melanogaster*, the underlying gene sets driving these patterns differ substantially. In *L. vannamei*, the mid-molt peak in younger gene expression is primarily driven by genes involved in cuticle development, whereas in *Drosophila*, the same pattern is largely associated with the expression of reproductive genes. Interestingly, in *Drosophila*, gene modules involved with cuticle development are also composed of younger phylostrata, but they are predominantly expressed earlier during pupariation, rather than in mid-molt as in *L. vannamei*. This heterochrony in cuticle gene expression is likely related to the evolution of the puparium in cyclorrhaphous flies, which retain the larval cuticle as a protective case rather than shedding it during ecdysis. Heterochrony in cuticle development and maturation has been linked to adaptation in other arthropods (Elias-Neto et al. 2014; Falcon et al. 2019), which suggests that timing of cuticle formation can evolve in response to different life history and ecological needs.

Although molting is regulated by conserved hormonal pathways, our findings suggest that it coordinates both deeply conserved and recently evolved gene modules. The precise coordination of these gene modules reflects a regulatory flexibility that enables molting to accommodate lineage-specific physiological and developmental requirements. It is likely that the regulatory architecture underlying molting allows for coexpression of genes not directly tied to the molting process itself but essential for broad physiological support and reconstructing the complex structure of the exoskeleton. Since the exoskeleton forms the primary interface between the organism and its environment, environmental pressures may contribute to its morphology and the gene expression programs involved in its formation. Divergence time and environmental heterogeneity have been shown to influence convergence in both gene expression and morphological traits (Buckley et al. 2009; Chaturvedi et al. 2022; Boisseau et al. 2025). Testing whether ecological niche similarity predicts convergence in ecdysis gene expression would therefore be an interesting task for future work.

By uncovering both conserved and lineage-specific components of molting gene expression, this study provides evidence for both evolutionary conservation and divergence of this deeply shared developmental process. This conservation of the molting program appears to extend to pupariation. These findings suggest that molting may involve a broader range of developmental processes than its classical definition of periodic shedding of the exoskeleton, which may encompass diverse molting strategies exhibited by Arthropods, ranging from puparium formation to, potentially, apolysis without ecdysis, as observed in paedomorphic insects such as Strepsipterans (Kathirithamby et al. 1984; Manfredini et al. 2007). In a broader perspective, molting can be viewed as a developmental process that enables growth and morphological transformation through *shedding or reconstruction* of the exoskeleton. Overall, our findings contribute to a broader understanding of the evolutionary dynamics underlying molting and highlight the modular architecture of the molting program, which may be central to understanding how the vast diversity of arthropod species has adapted their postembryonic development to different ecological and life-history contexts.

## Materials and Methods

### Data Pre-processing

Table 1 summarizes the RNA sequencing (RNA-seq) data used in this study. Additional time-series datasets were retrieved for *D. melanogaster* from (Ozerova et al. 2025) and for *L. vannamei* from Gao et al. 2015 (Table S1). The quality of the reads was checked using FastQC v.0.11.2 (Andrews 2010), and adapter trimming and quality control were performed using Trimmomatic v. 0.36 (Bolger et al. 2014) (Table S5). Trimmed reads were pseudo-aligned against the respective transcriptome of each species using kallisto v0.44.0 (Bray et al. 2016). Transcriptome and annotation files were retrieved from National Center for Biotechnology Information (NCBI) databases (Sayers et al. 2024). The quality and completeness of the genome assembly of each species were assessed with Benchmarking Universal Single-Copy Orthologs (BUSCO) using the Arthropoda lineage dataset (odb10) as reference (Simão et al. 2015) through the A3CAT database (Feron and Waterhouse 2022). All assemblies used in this study have a BUSCO score of more than 90% (Table S6). Transcript-level expression was imported into R v4.2.2 and summarised to the gene level using the R/tximport v1.10.1 (Soneson et al. 2016).

### Construction of Orthogroup Gene Expression Matrix

To examine gene expression patterns of molting stages between groups of species, orthologous genes (orthogroups) were identified using Orthologer v3.0.2 with default parameters (Kuznetsov et al. 2023) after filtering the proteomes for the single longest protein-coding isoform of each gene. Orthology inference was performed using a broader dataset including additional crustacean species (*Penaeus japonicus* and *Eriocheir sinensis*) beyond the five species in this study in order to improve orthology assignment associated with limited taxonomic sampling. The cross-species expression matrix was constructed from orthogroups shared across all five species and was not restricted to strict one-to-one orthologs. Because some orthogroups contained multiple genes per species, a single representative gene per species was selected based on the highest mean expression across samples, generating a read count matrix of 5,543 orthogroups identified across the five species. This orthogroup matrix was used exclusively for PCA and clustering analysis, whereas differential expression analyses were performed independently within each species using the original gene-level datasets. To assess the robustness of the representative gene selection strategy, we also used an alternative orthogroup matrix in which a random representative gene was selected per orthogroup. This produced similar overall PCA patterns, indicating that the major biological signal is robust to the choice of orthogroup representative (Fig. S6).

### Principal Component Analysis

Prior to conducting cross-species PCA, PCA was first performed separately for each species to assess sample clustering of molting stages within each dataset. Combined raw read counts from the 5,543 orthogroups identified across the five species were transformed using variance stabilizing transformation (VST function) implemented in DESeq2 (Love et al. 2014). This transformation generated constant variances across all read counts, creating a homoscedastic dataset. To account for the effect of species on gene expression, we included species as an explanatory variable in the model when constructing the read counts matrix. A PCA was conducted to examine the overall gene expression pattern of the dataset using the PCAtools R package (Blighe and Lun 2022) on the VST RNA-seq counts. The top 10 PCs were selected, and individual eigenvalues were tested for correlation with the traits of interest: taxonomic group, molting stage, and species.

### Differential Gene Expression Analysis

Differential gene expression analyses between premolt and postmolt stages were performed individually per species using DESeq2 (Love et al. 2014), with *P*-values adjusted using the Benjamini–Hochberg (Benjamini and Hochberg 1995) procedure to control for false discovery rate. An adjusted *P*-value of 0.05 was used as the threshold for identifying DEGs. For simplicity, wandering and prepupal stages of the larval–prepupal molt are referred to as simply premolt and postmolt, respectively. The resulting DEGs from each species were grouped based on orthology of DEGs into the following categories: species-specific genes (differential expression not shared with any other species), Pancrustacea shared genes (differential expression in at least one insect and one malacostracan), class-specific genes restricted to either class Insecta or Malacostraca (differential expression in at least two species per group), and genes which have differential expression exclusive to metamorphic or to nonmetamorphic samples. For each DEG, we also assessed whether its corresponding orthologous group, irrespective of differential expression, was restricted to a single lineage (insect-only or malacostracan-only), shared between insects and malacostracans (Pancrustacea-shared), or lacked detectable orthologs in other taxa. We note that genes classified as having no detectable orthologs may nevertheless have orthologs in additional closely related taxa not included in the current comparison, such as other Dipterans. Thus, a species-specific DEG may either represent a gene with no detectable ortholog in the present dataset, or the only differentially expressed member of an orthogroup otherwise shared across multiple species in the study.

### Clustering and Functional Analysis

Clust (Abu-Jamous and Kelly 2018) was used to identify and extract distinct groups of genes displaying correlated expression patterns across all samples. Unlike standard clustering approaches, Clust does not require prespecifying a fixed number of clusters. Briefly, Clust applies k-means clustering using a range of k-values to generate an initial set of seed clusters, which are subsequently refined using M–N scatter plots to identify large, nonoverlapping clusters with low dispersion. As such, the final number of clusters is determined dynamically by the data rather than fixed a priori. We used the default of Clust which filters out genes with flat expression profiles prior to analysis. Several cluster tightness values ranging from 0.1 to 1 were evaluated. Smaller tightness values produce larger and more diffuse clusters, whereas larger values produce smaller and more tightly coexpressed clusters. A tightness value of 1 was selected for this study as it produced clusters with minimal dispersion and clear expression patterns. Gene Ontology enrichment analysis (Ashburner et al. 2000) of genes belonging to each cluster was performed using ClusterProfiler (Wu et al. 2021). Putative cuticular protein families were identified using CutProtFam-Pred (Ioannidou et al. 2014), which uses Hidden Markov Models based on CuticleDB (Magkrioti et al. 2004) to detect and classify cuticular protein families based on conserved sequence motifs, including cuticular protein subfamilies Rebers and Riddiford type 1 (RR-1) and type 2 (RR-2), as well as additional families such as tweedle and cuticular protein analogous to peritrophins types 1 and 3 (CPAP1 and CPAP3).

### Transcriptome Age Analysis and Phylostratigraphy

To examine the conservation of gene expression across different timepoints of molting, phylostratigraphy analysis was used to estimate the relative transcriptome age of the time-series samples (Table S1) (Domazet-Lošo and Tautz 2010). VST-transformed counts were generated using DESeq2 (Love et al. 2014), and mean expression values for each stage were calculated. The resulting gene expression matrix was then clustered using Clust (Abu-Jamous and Kelly 2018), and the phylostratum composition for each cluster was determined by using GenEra v1.4.0 (Barrera-Redondo et al. 2023). All genes of a given target genome were queried against a protein database built from eukaryotic reference genomes retrieved from NCBI using DIAMOND *blastp* in more-sensitive mode with an *e*-value threshold of 10^−5^ (Buchfink et al. 2015). *Blastp* hits were considered homologs of the query protein and were used to determine the phylostrata of the gene.

The TAI of RNA-seq samples was calculated as described previously (Domazet-Lošo and Tautz 2010) using the *myTAI* (v2.0.0) (Drost et al. 2018) package in R (v4.3.2). TAI values were computed as the weighted mean of the phylostratum rank of a given gene based on its expression level in the transcriptome of an RNA-seq sample. VST transformed gene expression was used, which like log-transform removes the impact of extreme values (see J. Piasecka et al. 2013; Liu and Robinson-Rechavi 2018). A high TAI value indicates a younger transcriptome age, whereas a lower TAI corresponds to an older transcriptome. TAI profile standard deviations were estimated from 10,000 bootstrap permutations using the bootMatrix() function implemented in *myTAI* (v2.0.0) (Drost et al. 2018) and displayed as a shaded area in each TAI profile plots. Statistical significance of the TAI profile was determined through the flat line test, a permutation-based test implemented in *myTAI* (v2.0.0) (Drost et al. 2018). This test assesses whether the overall TAI trajectory deviates from a random expectation and therefore evaluates the global structure of transcriptome age dynamics across the molting process rather than pairwise differences between individual stages.

## Supplementary Material

evag212_Supplementary_Data

## Data Availability

All data used in this study were publicly available datasets retrieved from NCBI with the following accession numbers. *D. melanogaster* (PRJNA75285, PRJNA1124160), *Z. cucurbitae* (PRJEB36344, PRJNA562032), *B. dorsalis* (PRJNA524281), *L. vannamei* (PRJNA288849), and *S. paramamosain* (PRJNA687923). Additional supplementary files and scripts used in this study are available at Zenodo: *10.5281/zenodo.17784461*.
